# Experiences of professional public health advocacy in the UK health and social care system: a qualitative survey study [using the Theoretical Domains Framework]

**DOI:** 10.1093/pubmed/fdag035

**Published:** 2026-05-13

**Authors:** Kaat Marynissen, Akshay Pabary, Malcolm Moffat

**Affiliations:** Population Health Sciences Institute, Baddiley-Clark Building Richardson Road, Newcastle University, Newcastle upon Tyne, NE2 4AX, UK; Population Health Sciences Institute, Baddiley-Clark Building Richardson Road, Newcastle University, Newcastle upon Tyne, NE2 4AX, UK; Population Health Sciences Institute, Baddiley-Clark Building Richardson Road, Newcastle University, Newcastle upon Tyne, NE2 4AX, UK

**Keywords:** work environment, management and policy, public health, advocacy

## Abstract

**Background:**

Advocacy of public health principles (PHP) is crucial to tackling health inequities. Advocacy skills are therefore a core competency of Public Health speciality training in the UK. A survey of UK-trained specialist registrars found that the perceived ability to advocate for PHP varied significantly across work settings. This study explores barriers and enablers to PHP advocacy across workplaces, offering insights into how advocacy can be better supported in the UK public health landscape.

**Methods:**

An online survey was sent to UK-based public health specialty trainees, consultants, and public health practitioners on the portfolio route to consultant practice currently. Free-text survey responses were analysed using template analysis and the Theoretical Domains Framework.

**Results:**

Sixty-four respondents completed the survey. This study found that role clarity, organizational support, and advocacy skills significantly affect public health specialists’ ability to advocate. Barriers included unclear responsibilities, fear of consequences, resource constraints, and inadequate training and mentorship. Supportive leadership and shared values facilitated success.

**Conclusions:**

To promote advocacy, the Faculty of Public Health should ensure specialists are positioned as autonomous experts, address biases in recruitment, and integrate advocacy skills further into training. Organizations must evaluate how their structure, culture, and leadership support public health advocacy.

## Background

Public health principles (PHP), defined as ‘social justice and equity, promoting and protecting better health for all [with] a resolute focus on tackling inequalities in health, including those driven by racism and discrimination’, ^1^ are central to tackling pernicious problems in health and care. Publications such as the 2010 Marmot Review^2^ highlighted how stark differences in health outcomes between communities are largely determined by social factors. They also demonstrate how advocacy of PHP can influence health policy; the Marmot Review led to the founding of ‘Marmot Places,’ where local authorities address social determinants of health through interventions prioritizing health equity.^3^ Despite this, the social gradient of health outcomes across the UK remains stark.^4–7^ With renewed focus on prevention in governmental directives,^8^ it is vital to place PHP at the centre of policy discussions.

Advocacy, defined here as the championing of PHP within PH specialists’ everyday work, is central to effecting policy change. While advocacy has been explored among PH practitioners,^9^ it remains under-examined among specialists. Public health specialists (Specialty Registrars (StRs) during training and Consultants in Public Health post-training) work in various organizations, including government bodies, the National Health Service (NHS), and the voluntary, community and social enterprise sector. This, alongside trainee recruitment from clinical and non-clinical backgrounds, results in a workforce with broad experience situated in many sectors influencing population health. While this can be an asset, there is little understanding of how the ability to advocate for PHP varies across organizations. A lack of studies exploring specialists’ perceived ability to advocate for PHP across their diverse workplaces constitutes a research gap. Furthermore, while the Theoretical Domains Framework (TDF) has been used to determine factors influencing behaviour among healthcare workers,^10^ it has not been applied in relation to advocacy. As a result, this study aims to utilize the TDF to explore the barriers and enablers to PHP advocacy across workplaces, providing insights into how advocacy can be strengthened to help specialists tackle health inequities effectively.

## Methods

### Survey

An online survey was distributed to public health specialists across the UK via the Jisc Online Surveys platform. Respondents were eligible to participate if they were a specialty trainee, consultant, or public health practitioner on the portfolio route currently working in the UK. Survey questions focussed on advocacy, defined as the ‘act of persuading or arguing in support of a specific cause, policy, idea or set of values’,^11^, p.177 rather than activism, which often refers to ‘the use of vigorous campaigning to bring about political or social change’.^12^

Survey questions were developed in collaboration with Specialty Registrar Committee (SRC) colleagues and based on findings of a previous SRC survey completed by StRs only. Questions considered barriers and enablers across work settings and explored potential challenges due to employment/placements, knowledge/confidence and practical issues (see Appendix 1). A pilot version was tested in March 2025 and revised based on feedback, including clarifying definitions of advocacy and PHP and wording of certain questions. The final survey was distributed from March to May 2025.

### Analysis

Free-text survey responses were analysed using template analysis, a framework analysis method (FAM). Within template analysis data is codified according to codes identified prior to analysis.^13^ Codes were drawn from the Theoretical Domains Framework (TDF), a tool developed to provide a comprehensive, theory-based approach to identifying determinants of behaviour.^14^ Responses were categorized by TDF domains and subsequently organized into themes, utilizing NVivo 15 software. Two researchers (KM and AP) read and independently coded all free-text responses. Coding was compared for consistency and discrepancies were resolved via discussion with the third author (MM). As is usual with FAMs, data interpretation was refined throughout the writing process.^13^ Ethical approval was granted by Newcastle University Ethics Committee in February 2025 (Ref: 56225/2023).

## Results

### Respondent demographics

Sixty-four respondents completed the survey. Most (46.9%) had entered public health via the national training scheme from a medical or dental background, while 42.2% had undertaken the training scheme from a non-medical background and the remaining 10.9% via the portfolio route. Just over half (51.6%) of respondents were in the latter stages of training (Speciality Training grades 3–5) and almost a quarter (23.4%) were Consultants in Public Health. Respondent demographics are shown in Table 1.

**Table 1:** Participant characteristics (n = 64).

***Route into public health***		***n (%)***
	Portfolio route	7 (10.9)
	Training scheme (medical or dental background)	30 (46.9)
	Training scheme (non-medical background)	27 (42.2)
***Current training/competency level***		
	PH practitioner	5 (7.8)
	Consultant < 5 years post-CCT	8 (12.5)
	Consultant > 5 years post-CCT	7 (10.9)
	ST1	2 (3.1)
	ST2	9 (14.1)
	ST3	11 (17.2)
	ST4	12 (18.8)
	ST5	10 (15.6)
		
	Consultant	15 (23.4)
	ST 1–2	11 (17.2)
	ST 3–5	33 (51.6)
***Current training/work location***		
	North East	14 (21.9)
	London	9 (14.1)
	Yorkshire and Humber	5 (7.8)
	West Midlands	5 (7.8)
	Thames Valley	5 (7.8)
	North West	4 (6.3)
	Scotland	3 (4.7)
	East of England	3 (4.7)
	South West	3 (4.7)
	Wessex	3 (4.7)
	Wales	2 (3.1)
	East Midlands	2 (3.1)
	Kent, Surrey and Sussex	2 (3.1)
	Defence Medical Services	2 (3.1)
	Northern Ireland	1 (1.6)
	Prefer not to say	1 (1.6)
***Gender identity***		
	Female	41 (64.1)
	Male	16 (25.0)
	Non-binary	2 (3.1)
	Prefer not to say	5 (7.8)
***Age***		
	26–30	8 (12.5)
	31–35	17 (26.6)
	36–40	13 (20.3)
	41–45	11 (17.2)
	46–50	6 (9.4)
	51–55	4 (6.3)
	56–60	1 (1.6)
	61+	1 (1.6)
	Prefer not to say	3 (4.7)
***Ethnicity***		
	White	48 (75.0)
	Asian or Asian British	6 (9.4)
	Mixed or multiple ethnic groups	4 (6.3)
	Prefer not to say	6 (9.4)

### Identified themes

Most free-text survey responses fell into three domains of the TDF: social/professional role and identity, environmental context and resources, and social influences. The remainder of the responses was captured in the domains of skills, goals, knowledge, beliefs about consequences, beliefs about capabilities and emotion. Supporting quotes for the themes discussed below are found in Table 2, with further quotes provided in Appendix 2.

**Table 2:** Supporting quotes for identified themes.

***Social and professional role***	
Advocacy as integral to professional role	‘I feel that it’s part of my professional duty to advocate for public health principles […] I’ve never come across a situation where anyone has tried to stop me doing it—I assume that they expect me to do it as part of my professional role.’ (P26, Consultant (medical or dental background), > 5 yrs post-CCT) ‘Advocacy within my role in international organization work was core to the work and clearly encouraged.’ (P45, Registrar (medical or dental background), ST2)
Challenges of public health role being perceived differently by others	‘[T]here are advocacy organizations that I would want to approach but feel that I may be frowned upon as as [sic] a public health representative because we are (or at least often perceived to be) somewhat aligned to the state or to government decision making. On the other hand, however, I feel that within the work/professional settings and communities which I have access to (local authority public health team, faculty of public health, BMA) that some of the causes I want to advocate for would be seen as politically risky and “too far to the left”’ (P23, Registrar (medical or dental background), ST1) ‘[A barrier to advocacy is] [l]ack of authority as a trainee’ (P4, Consultant (non-medical background), < 5 yrs post-CCT)
Influence of organizational priorities: local authorities	‘In the local authority […] everyone had a good understanding of public health principles, so advocacy with external stakeholders was encouraged and considered part of the job’ (P10, Registrar (medical or dental background), ST2) ‘Local authority: [I] work in a Marmot city, so advocacy for public health principles is discussed frequently.’ (P14, Registrar (non-medical background), ST2)
Influence of organizational priorities: NHS trusts	‘NHS—More limited scope. Genuine interest and appetite but rhetoric (i.e. shift to prevention, tackling inequalities) can be difficult to turn into reality at scale due to focus on immediate pressure and shorter term planning/outcomes.’ (P56, Consultant (portfolio, non-medical background), < 5 yrs post-CCT) ‘Similarly, NHS trusts have their own hierarchies and priorities which may not always make PH a priority. It seems if you can create links between public health principles having mutual benefits to their own metrics and priorities there are also opportunities for advocacy.’ (P24, Registrar (medical or dental background), ST2)
Influence of organizational priorities: central government organizations	‘Central government is heavily constrained by delivery focus, policy, protocol, programme for government, and ministerial priorities.’ (P3, Registrar (medical or dental background), ST4) ‘My experience of DHSC is that public health principles are very much secondary to ministerial priorities, and many civil servants see their primary responsibility as sophistry in support of ministerial priorities and public health values are rapidly reduced to rhetorical devices.’ (P9, Consultant (non-medical background), < 5 yrs post-CCT)
Influence of organizational priorities: academia	‘In academia, I think sometimes the focus on principles can hinder practical progress—there is a certain amount purism that is very admirable but does not always translate to on the ground action in public health practice.’ (P11, Consultant (non-medical background), < 5 yrs post-CCT) ‘[A]cademic public health can often take a narrow viewpoint due to the specialized nature of research, and teams are often lacking in diversity which is likely to have an impact given how research questions are identified, prioritized, and addressed.’ (P16, Registrar (non-medical background), ST3)
Influence of protocol-driven or operational roles	‘My work in UKHSA was reactive and I do feel that some of their policies do not consider equity/health inequalities especially strongly. As it is quite protocol driven I am not sure I felt able to advocate differently.’ (P8, Registrar (medical or dental background), ST3) ‘In UKSHA I felt much more pressure to focus on the job in front of me rather than making change in any meaningful way’ (P11, Consultant (non-medical background), < 5 yrs post-CCT) ‘Health protection teams are disconnected—they have become a tick box exercise with no head space for wider public health considerations’ (P44, Registrar (medical or dental background), ST5)
***Environmental context and resources***	
Impact of financial constraints	‘[Barriers include] loss of grant funding to other council budgets, specialist analytical staff lost into corporate functions or left due to lack of value of technical skills, ph having to take on management of other council functions’ (P33, Consultant (medical or dental background), > 5 yrs post-CCT) ‘Advocacy for local public health measures is impacted by the resourcing of local systems. With most teams having made substantial cuts in recent years to services, it can be difficult to advocate or initiate system action knowing that resources are already being diverted away from essential public health action’ (P40, Registrar (medical or dental background), ST5)
Need to project awareness of resource constraints	‘In local authorities and NHS trusts, you have to be somewhat politically savvy if you want to make change—if you advocate too strongly and idealistically, you will be dismissed as not understanding the practical constraints of the situation’ (P11, Consultant (non-medical background), < 5 yrs post-CCT)
Placement of PH practitioners within organizational structures	‘DsPH [Directors of Public Health] that are directly accountable to the Chief Executive Officer Are better able to influence I think than those that report to other Directors; these DsPH can directly shape the council strategy and advocate for Public Health independently of other directorates’ (P40, Registrar (medical or dental background), ST5)
Organizational culture, including political influence and alignment	‘Having an environment that is open to ideas and discussions is an important factor in promoting or allowing for advocacy in public health.’ (P32, Consultant (medical or dental background), > 5 yrs post-CCT) ‘The political environment of my local authority placement has allowed me to advocate for public health principles that I am interested in and care deeply about […] I am not sure this would have been well received or even allowed at different, adjacent councils with other political leanings.’ (P23, Registrar (medical or dental background), ST1) ‘In the LA our posts are politically restricted so we cant be publicly party political critical’ (P58, Consultant (non-medical background), > 5 yrs post-CCT) ‘The negative aspects are related to observed hesitation from colleagues to engage where the issue is politically sensitive.’ (P18, Registrar (non-medical background), ST2)
***Social influences***	
Need for supportive teams with aligned values	‘I also have well established activists in my public health team who I can look for their vocal, longstanding and admirable work.’ (P23, Registrar (medical or dental background), ST1) ‘As a team we share collective values and support each other in trying to implement these’ (P27, Consultant, (medical or dental background), > 5 yrs post-CCT) ‘[A challenge is] [l]ack of support from colleagues who don’t fight the fight with you.’ (P1, Registrar (non-medical background), ST4)
Transient nature of PH training as a barrier	‘The positive aspects is that the colleagues in the Public Health and communities team are tuned in and enthusiastic about everything to do with public health which makes it easier to advocate for public health principles’ (P18, Registrar (non-medical background), ST2) ‘generally not in organizations long enough to influence or advocate for long term change as relationship building and change takes time which on the training scheme we are short of.’ (P55, Registrar (medical or dental background), ST5) ‘short timeframes leading to not feeling embedded is a big issue’ (P17, Registrar (non-medical background), ST3)
Value of strong leadership, including challenges of leadership with a dismissive or negative view of PH	‘I think the facilitator here might have been my project supervisor who has been an articulate and [sic] enthusiastic advocate for the public health skillset (even though she is not public health trained) which has created equal enthusiasm and a respect for the expertise from the rest of the team’ (P42, Registrar (non-medical background), ST4) ‘Many senior public health figures seem not to understand the importance of agenda setting and wait for political direction on their agendas and remits.’ (P47, Registrar (medical or dental background), ST5) ‘I am no longer managed by a public health consultant, and my DD [deputy director] is a career civil servant who has been very negative about the profession. I have tried to advocate for public health and been told I am ‘undermining team cohesion’ and ‘impacting colleagues’ confidence’ when taking a public health approach.’ (P50, Practitioner (portfolio) (non-medical background))
Impact of bias within the training programme on leadership	‘I think the training programme has in recent years been guilty of differential treatment of registrars/favouring certain types of practitioners […] these issues also create problems in the types of leaders created and promoted’ (P40, Registrar (medical or dental background), ST5)
***Skills/Goals/Knowledge***	
Influence of skills on perceived ability to advocate	‘I feel that my experience and training has enabled me to advocate in all settings, whether people agree with me or not and whether my opinion is asked for’ (P54, Consultant (portfolio) (non-medical background), < 5 yrs post-CCT) ‘it would be nice to receive formal advocacy training.’ (P10, Registrar (medical or dental background), ST2) ‘Training programmes do not prepare registrars for working within these systems, instead putting the onus on StRs to “reflect on system leadership” with little guidance or tuition.’ (P40, Registrar (medical or dental background), ST5)
Importance of clear vision or goals	‘Enabling [factors]: role model, clarity of vision that included PH principles, being able to “sell” importance’ (P36, Registrar (medical or dental background), ST3)
Understanding of PH principles within a team	‘Generally, I think the pandemic gave a renewed focus on inequalities, racism, and the health of communities to a wider range of local authority partners and teams. Health genuinely became everyone’s business’ (P63, Registrar (non-medical background), ST1) ‘I note senior leaders understand the principles and are bought-in whereas more junior colleague civil servants do not have an in-depth understanding of health and public health principles in order to complete tasks in a meaningful way.’ (P51, Registrar (medical or dental background), ST4)
***Emotion***	
Fear of being viewed as a dissident, including potential career repercussions	‘I’ve worked in regional teams where I think there is real fear of being viewed as a dissident and the subsequent loss of power/influence/jobs.’ (P40, Registrar (medical or dental background), ST5) ‘there is a degree of personal challenges to advocacy and the position this can leave you in within a board’ (P45, Registrar (medical or dental background), ST2)
Personal discomfort born from confronting one’s own biases	‘Advocacy (and to a greater extent activism) requires a certain degree of personal discomfort, professional vulnerability/risk, cultural humility, and a continuing process of unlearning—these are things that senior public health figures and establishments have no appetite for.’ (P47, Registrar (medical or dental background), ST5)

#### Social and professional role

The importance of social/professional role and identity was one of the most widely reported themes. Respondents referred to advocacy as integral to their professional role and noted that where this perception was shared by others in the organization, their ability to advocate was greatly improved.

However, respondents’ role as perceived by others could also lead to tension and lack of clarity on what they were ‘allowed’ to do, which could leave them feeling isolated and unsupported. Another barrier to effective advocacy was a lack of authority, particularly during training.

As well as perceptions of professional role, respondents noted that their ability to advocate for PHP was influenced by the type of work they undertook, as well as organizational priorities. It was generally felt that local authorities were already ‘bought into’ and supportive of PHP, and advocacy was easier as a result.

NHS Trusts were seen as supportive of PHP in theory, but respondents noted that these were often de-prioritized in favour of acute clinical priorities. This was echoed by those who had worked in central government organizations such as the Department of Health and Social Care (DHSC), where PHP were felt to come second to ministerial priorities.

In academic spaces respondents reflected that while PHP were embraced, other elements of research such as academic rigour and a niche focus could hamper the actioning of these principles in practice. The equating of advocacy for principles with ability to translate those principles into action was also seen strongly within the ‘environmental context and resources’ domain explored below.

Respondents stated that workplaces with more protocol-driven or operational roles often did not allow them to engage with wider principles beyond the immediate scope of their role. This came through particularly strongly with health protection team roles within the UK Health Security Agency (UKHSA), which were felt to be disconnected from the wider PH system.

#### Environmental context and resources

The impact of financial constraints was considered a significant barrier to PH advocacy. Such constraints affected advocacy by forcing PH practitioners to take on other roles within an organization, which diluted their ability to action core public health functions, or by precluding advocacy for PH initiatives that were not perceived to be economically viable. Respondents stated the need to project awareness of resource constraints as part of effective advocacy, as a lack of awareness of practical constraints might lead to ideas being dismissed as being too idealistic.

As well as resource constraints, advocacy was impacted by where PH practitioners sat within organizational structures. Structural differences were noted to impact practitioner independence/autonomy, as well as their ability to influence. Besides resources and organizational structure, respondents felt that organizational culture (defined as ‘a collection of fundamental assumptions, norms, values, and shared conduct transmitted to newcomers’^15^, p.1) greatly influenced an ability to raise concerns. This was echoed within the *emotion* domain, where psychological safety was viewed as a key enabler of advocacy.

The importance of a supportive and open culture was further explored in the emphasis that respondents placed on political influences and alignment. This was largely stated in relation to explicitly political organizations such as local authorities. However, several respondents referred to the impact of politics in more general terms, including hesitancy from colleagues in tackling ‘politically sensitive’ topics.

#### Social influences

Similar to the need for political alignment, respondents’ consideration of social influences emphasized the importance of alignment within teams and between teams and senior leadership. Respondents felt more able to advocate in supportive teams with aligned values, or in teams led by role models. The transient nature of PH training was seen as a potential barrier to effective advocacy, as trainees’ position as an ‘outsider’ in most teams led to a reduced ability to influence colleagues.

Alongside supportive teams, strong leadership was seen as crucial to generating support for PHP within teams and at management level. Leadership could also be a significant barrier. Respondents articulated the challenges of leadership with a dismissive or negative view of PH, or those led by other principles such as the political directions of ministers. One respondent intimated that issues with leadership could originate from bias within the training programme, which they felt favoured certain kinds of practitioner, resulting in the kinds of leaders that were subsequently promoted.

#### Skills/goals/knowledge

The influence of skills on perceived ability to advocate within a workplace was variable. While some felt that their training/experience had equipped them with the skills to feel confident to advocate, others reflected on the need for more formal advocacy training and what they felt were shortcomings of the national training programme. In tandem with this, having clear visions or goals that include PHP within organizations were key to helping set strategic vision and ensuring buy in.

Finally, participants reflected on how an understanding of PHP within a team engendered enthusiasm for PH advocacy, while a lack of understanding could lead to ignorance of how or why advocacy of PHP should be undertaken.

#### Emotion

The need for psychological safety captured within the emotion domain has been described above. Other key themes centred on the fear of being viewed as a dissident and the potential repercussions this could have on one’s career. There were also reflections on how advocacy forces people to confront their own biases and undergo a process of unlearning, which can lead to personal discomfort that practitioners or organizations may be unwilling to engage with.

## Discussion

### Main findings of this study

This study identified key factors influencing public health specialists’ ability to advocate for PHP. Role clarity and authority were crucial; specialists felt empowered when advocacy was perceived as integral to their role and understanding of PHP within the team was high, while unclear boundaries or lack of authority hindered advocacy. Organizational setting played a major role - local authorities were considered more supportive of public health advocacy, while other settings often prioritized other factors. Such de-prioritization could translate into financial constraints and limited allocated resources, further constraining advocacy efforts. The structure and culture of the organization, including political alignment and seniority of PH specialists, also impacted advocacy success, while shared values and effective leadership were vital for support. Finally, training programs were sometimes criticized for leaving specialists unprepared for advocacy or for prioritizing certain kinds of practitioner.

### What is already known on this topic

Advocacy has been noted to play a key role in translating research into policy and practice, as well as engaging with interest groups and governments to affect change.^16^ Amongst health professionals, advocacy is often seen as a professional responsibility and even ethical obligation,^17^^,^^18^ and this extends to public health practice.^19^ Effective PH advocacy requires skill and strategic planning.^20^ Developing advocacy skills forms a core part of the PH specialist training curriculum,^21^ and an ability to advocate has been noted as one of the key ‘talents’ of public health ‘superheroes.’^22^ Despite this, it has remained what Chapman called a ‘Cinderella branch’^23^, p.361 of public health work; ‘incandescent during its limited hours on the stage, only to resume pumpkin status after midnight’.^23^, p.361 Identified barriers to advocacy include a lack of skills and experience resulting in a reluctance to engage with advocacy,^24^ a neoliberal political and economic zeitgeist that encourages individualism and minimizes the need for collective action or responsibility,^25^^,^^26^ political short-termism,^25^ and a focus on the biomedical model of health that prioritizes acute health services or technological treatments for disease over social determinants.^25^ Public health advocacy can also be deeply unpopular, with governments, corporations and even health authorities regarding it as unprofessional, or academics perceiving it as unscientific and biased.^19^

### What this study adds

This study is the first to explore how barriers and enablers to PH advocacy are enacted in the UK public health landscape. It complements a growing number of studies utilizing the TDF to explore public health topics^27^ by applying this framework in the methodologically novel context of PH practitioners’ behaviour around advocacy. It highlights the variation amongst workplaces in viewing PH specialists as either independent professionals or as service providers, and the tension that arises when an organization’s perception of PH specialists’ role differs from the specialist’s own view. Respondents felt frustrated in roles where their expertise was seen as subordinate to other organizational priorities. This echoes Tillmann et al.’s criticism of institutional restrictions preventing bold analysis of public health problems,^28^ suggesting that a lack of professional independence remains a persistent issue. Respondents reported that organizational structures and allocated resources often affected their ability to advocate for PHP. Barriers included an inability to translate principles into action due to constrained finances and organizational culture, including political alignment between PHP and the organization. The need for a supportive culture within organizations echoes findings within other healthcare teams, which show that the ability to ask questions and raise concerns is a key to high quality and safe practice.^29^ Our findings re-emphasize the impact of political and economic influences on the ability to advocate for PHP. This echoes known consequences of wider political and economic influences, such as the impact of fiscal austerity on public health expenditure in England.^30^ The need to engage with these forces for effective advocacy to take place supports the view that engaging with public health policy is an inherently political act.^31^^,^^32^ Another key insight is the importance of aligned values for effective advocacy, both within and between teams and senior leadership. Ensuring this alignment includes creating systems that allow leadership to be representative of the wider workforce and the communities it serves. A recent analysis of PH specialty training identified bias within recruitment processes, with certain demographics such as black and Asian candidates significantly less likely to secure a training post than white and other ethnic minority candidates.^33^ Differential attainment persists within training, with older candidates, black and Asian candidates and those from a professional background other than medicine being significantly less likely to pass professional examinations on first attempt.^34^ Where systematic differential attainment is identified it must be addressed if we are to guarantee that training and therefore leadership positions do not favour one kind of practitioner. The need for representative leadership acknowledges that specialists’ experiences of organizational factors are modulated by intersecting identities, including ethnicity or professional background, as outlined in Crenshaw’s work on intersectionality.^35^ Finally, this paper highlights that PH specialists’ needs are similar to those amongst practitioners as detailed by Sykes et al., including the need for specific advocacy skills, the role that mentorship and communities of practice can play in creating the psychological safety specialists need to advocate, and the need for an improved understanding of PHP/advocacy within organizational governance structures.^9^ Respondents emphasized that the consequences of challenging the status quo can be a powerful deterrent to advocacy, supporting the need for coalitions with like-minded individuals and organizations.^36^

Based on these findings, our most urgent recommendation is that, within policy, the FPH support PH specialists’ position within organizations as that of an autonomous ‘critical friend’; able to provide independent expertise in line with PHP as opposed to political or economic priorities. We also call upon the FPH to address known inequalities in recruitment to specialist training and differential attainment in professional examinations. In PH practice, we recommend embedding advocacy skills more broadly in the PH curriculum, and that organizations critically assess how they can foster a culture, structure and leadership that allows for PHP advocacy. This is especially key in healthcare-oriented services such as the NHS/UKHSA, where generating an understanding of PHP and their relevance can be more challenging. While we acknowledge the complexity involved in implementing systemic changes, they are crucial to ensuring PH specialists can champion equitable PH initiatives that engage directly with health inequalities. Within research, we encourage greater academic interest in how advocacy is or isn’t enacted across PH organizations, including developing insights from this paper through more in-depth qualitative work with PH specialists via focus groups and interviews.

### Limitations of this study

Limitations of this study include the limited number of respondents relative to the entire UK PH specialist workforce and an over-representation of one geographic area (the North East), which constrains the generalizability of our findings. The under-representation of some placements/settings mean that insights have come from a small number of individuals and may not be representative across the wider workforce. Due to the ‘opt-in’ nature of the study there is a potential for self-selection bias, as well as self-report bias when submitting responses. The nature of the survey means it provided an oversight of key issues but did not afford the opportunity to interrogate responses in-depth in the same way as methods such as interview. Furthermore, the use of a predefined theoretical framework may have limited novel insights which were not easily categorized in existing TDF domains. The wording of survey questions may also have weighted responses towards logistical barriers to advocacy in workplaces and away from domains focussing on emotional contexts. This informed our recommendation above to continue developing insights with in-depth qualitative approaches such as interviews which may capture this better.

## Conclusion

Perceptions of public health specialists’ roles, organizational structure, culture, value alignment, knowledge of PHP, and confidence in advocating without fear of retribution all influence the success of public health advocacy across UK work settings. To promote advocacy, the FPH should ensure specialists are positioned as autonomous experts, address biases in recruitment, and integrate advocacy skills into training. Organizations must also evaluate how their structure, culture, and leadership support public health advocacy.

## Supplementary Material

JPH_Marynissen_et_al_appendix_1_survey_questions_fdag035

JPH_Marynissen_et_al_appendix_2_supplementary_quotes_fdag035

## Data Availability

The data that informed this paper is available from the authors upon reasonable request.

## Notes

**Kaat Marynissen**, Specialist Registrar in Public Health

**Akshay Pabary**, Specialist Registrar in Public Health

**Malcolm Moffat**, Consultant in Public Health

## References

[1] Faculty of Public Health . What is Public Health? 2025 [cited 2025 July 28]. https://www.fph.org.uk/what-is-public-health/.

[2] Marmot M . Fair Society, Healthy Lives: The Marmot Review, London: Institute of Health Equity, 2010.

[3] Institute of Health Equity . What is a Marmot Place? 2025 [cited 2025 Aug 01]. https://www.instituteofhealthequity.org/taking-action/marmot-places#:~:text=What%20is%20a%20Marmot%20Place%3F%20A%20Marmot%20Place,action%20to%20improve%20health%20and%20reduce%20health%20inequalities

[4] Marmot M, Allen J, Boyce T et al. *Health Equity in England: The Marmot Review 10 Years on*. London: Institute of Health Equity, 2020, 10.1136/bmj.m693 32094110

[5] Fenton L, Wyper GM, McCartney G et al. Socioeconomic inequality in recent adverse all-cause mortality trends in Scotland. J Epidemiol Community Health 2019;73:971–4. 10.1136/jech-2019-212300 31326891 PMC6817697

[6] Atcheson R, Laverty C. *Health Inequalities: Annual Report 2024*. Belfast: The Northern Ireland Statistics and Research Agency (NISRA), 2024.

[7] Perks W . Wellbeing of Wales, 2023. 2023 [cited 2025 Aug 05]. https://www.gov.wales/wellbeing-wales-2023-healthier-wales-html

[8] Department of Health and Social Care . Fit for the Future: 10 Year Health Plan for England. UK Government, 2025.

[9] Sykes S, Watkins M, Wills J. Public health practitioners as policy advocates: skills, attributes and development needs. Health Promot Int. 2023;38:daad102. 10.1093/heapro/daad102 37703396 PMC10499303

[10] Wong E, Mavondo F, Horvat L et al. Healthcare professionals’ perspective on delivering personalised and holistic care: using the theoretical domains framework. BMC Health Serv Res 2022;22:281. 10.1186/s12913-022-07630-1 35232432 PMC8887936

[11] Cox R, Pezzullo PC. *Environmental Communication and the Public Sphere*, 4th edn. Los Angeles: SAGE, 2016.

[12] Oxford English Dictionary . Activism. 2025 [cited 2025 July 28]. https://www.oed.com/dictionary/activism_n?tl=true

[13] Klingberg S, Stalmeijer RE, Varpio L. Using framework analysis methods for qualitative research: AMEE guide No. 164. Med Teach 2024;46:603–10. 10.1080/0142159X.2023.2259073 37734451

[14] Atkins L, Francis J, Islam R et al. A guide to using the theoretical domains framework of behaviour change to investigate implementation problems. Implement Sci 2017;12:77. 10.1186/s13012-017-0605-9 28637486 PMC5480145

[15] Tadesse Bogale A, Debela K. Organizational culture: a systematic review. Cogent Bus Manag 2024;11:2340129. 10.1080/23311975.2024.2340129

[16] Chapman S . Advocacy in public health: roles and challenges. Int J Epidemiol 2001;30:1226–32. 10.1093/ije/30.6.1226 11821312

[17] Olatunbosun C, Wilby KJ. Advocacy as a professional responsibility. Can Pharm J (Ott) 2022;155:298–301. 10.1177/17151635221125782 36386601 PMC9647394

[18] Wynia MK, Peek ME, Heisler M. When are health professionals ethically obligated to engage in public advocacy? Lancet. 2025;406:211–4. 10.1016/S0140-6736(25)01378-9 40639386

[19] Hancock T . Advocacy: it’s not a dirty word, it’sa duty. Can J Public Health 2015;106:e86–8. 10.17269/CJPH.106.5094 26125246 PMC6972452

[20] van Oort B, van 't Riet H, Parejo Pagador A et al. Understanding the what, how, and why in advocacy: assessing the applicability of participatory process evaluation methodology in an advocacy context. Evaluation. 2023;29:509–27. 10.1177/13563890231200057

[21] Faculty of Public Health . *Public Health Specialty Training Curriculum 2022*, 2022 [cited 2025 July 28]. https://www.fph.org.uk/media/4ojeo5bk/public-health-curriculum-2022-v13_final.pdf

[22] Day M, Shickle D, Smith K et al. Training public health superheroes: five talents for public health leadership. J Public Health (Oxf) 2014;36:552–61. 10.1093/pubmed/fdu004 24514154

[23] Chapman S . Advocacy for public health: a primer. J Epidemiol Public Health 2004;58:361–5. 10.1136/jech.2003.018051 PMC1732774 15082730

[24] Blenner SR, Lang CM, Prelip ML. Shifting the culture around public health advocacy: training future public health professionals to be effective agents of change. Health Promot Pract 2017;18:785–8. 10.1177/1524839917726764 28891348

[25] Farrer L, Marinetti C, Cavaco YK et al. Advocacy for health equity: a synthesis review. Milbank Q 2015;93:392–437. 10.1111/1468-0009.12112 26044634 PMC4462882

[26] Cohen BE, Marshall SG. Does public health advocacy seek to redress health inequities? A scoping review. Health Soc Care Community 2017;25:309–28. 10.1111/hsc.12320 26749000

[27] Zhou Y, Huang Y, Wang Y et al. Theoretical domains framework: a bibliometric and visualization analysis from 2005-2023. J Multidiscip Healthc 2024;17:4055–69. 10.2147/JMDH.S470223 39188813 PMC11345462

[28] Tillmann T, Baker P, Crocker-Buque T et al. Shortage of public health independence and advocacy in the UK. Lancet. 2014;383:213. 10.1016/S0140-6736(14)60064-7 24439730

[29] Shankar R, Devi F, Mukhopadhyay A. The role of leadership in fostering psychological safety in healthcare: protocol for a systematic review of qualitative studies. Int J Qual. Methods. 2025;24:1–6. 10.1177/16094069251333895

[30] Stokes J, Bower P, Guthrie B et al. Cuts to local government spending, multimorbidity and health-related quality of life: a longitudinal ecological study in England. Lancet Reg Health Eur 2022;19:100436. 10.1016/j.lanepe.2022.100436 36039277 PMC9417904

[31] Greer SL, Bekker M, De Leeuw E et al. Policy, politics and public health. Eur J Public Health 2017;27:40–3. 10.1093/eurpub/ckx152 29028231

[32] Coughlan J , Haywood L, Collonnaz M. You cannot practice public health without engaging in politics. 2021 [cited 2025 Aug 12]. https://blogs.bmj.com/bmj/2021/03/29/you-cannot-practice-public-health-without-engaging-in-politics/

[33] Bury F, Rao M, Pinder R. Differential attainment in public health specialty training recruitment in the United Kingdom: an observational analysis of applicants from 2018 to 2020. J Public Health (Oxf) 2023;45:330–7. 10.1093/pubmed/fdac122 36335426 PMC10273379

[34] Fardon R, Vusirikala A, Latif S, Chappel D . Equality, diversity and inclusion in the Membership of the Faculty of Public Health Examinations. London: Faculty of Public Health, 2024.

[35] Crenshaw K . Mapping the margins: intersectionality, identity politics, and violence against women of color. SLR. 1991;43:1241–99. 10.2307/1229039

[36] Murray J , Leigh-Hunt N. Training to be unpopular: five short steps to becoming a public health advocate. 2019 [cited 2025 Aug 12]. https://blogs.bmj.com/bmj/2019/04/29/training-to-be-unpopular-five-short-steps-to-becoming-a-public-health-advocate/

