# Supplementary material for: Experiences of professional public health advocacy in the UK health and social care system: a qualitative survey study [using the Theoretical Domains Framework]
Source: J Public Health (Oxf). 2026 May 13;48(2):610–9. doi: 10.1093/pubmed/fdag035 (PMC13223560; doi:10.1093/pubmed/fdag035)
Supplement: JPH_Marynissen_et_al_appendix_1_survey_questions_fdag035 [file jph_marynissen_et_al_appendix_1_survey_questions_fdag035.docx]

Appendix 1: Survey questions used in final survey

1. What is your current role in Public Health? (*free text*)

2. What has been your route into Public Health? (*dropdown, single selection allowed*)

Training scheme (medical or dental background)

Training scheme (non-medical background)

Portfolio route (medical or dental background)

Portfolio route (non-medical background)

Other (please see Q3)

3. If you answered 'other' please expand below (*free text*)

4. What is your current Speciality Training (ST) level? (*dropdown, single selection allowed*)

ST1

ST2

ST3

ST4

ST5

PH practitioner (portfolio scheme)

Consultant <5yrs post-CCT

Consultant >5yrs post-CCT

Other (please see Q5)

5. If you answered 'other' please expand below (*free text*)

6. Whereabouts in the UK are you training/working? (*dropdown, single selection allowed*)

Defence Medical Services

East Midlands

East of England

Kent, Surrey and Sussex

London

North East

North West

Northern Ireland

Scotland

South West

Thames Valley

Wales

Wessex

West Midlands

Yorkshire and Humber

Prefer not to say

7. What type of employment contract are you currently training/working under? (*dropdown, single selection allowed*)

Training placement

Employed (fixed term)

Employed (permanent)

Locum / self-employed

8. Please indicate all settings you have worked in as a public health professional (*dropdown, multiple selections allowed*)

Local authority

NHS Trust

NHS England - regional

NHS England - national

OHID (or equivalent) - regional

OHID (or equivalent) - national

DHSC (or equivalent) - regional

DHSC (or equivalent) - national

UKHSA (or equivalent) - regional

UKHSA (or equivalent) - national

Other regional (e.g. TfL, NWAS)

International organisation (e.g. WHO)

Voluntary, Community and Social Enterprise (VCSE)

Academia

Other (please see Q9)

9. If you answered 'other' please expand below (*free text*)

This survey defines public health principles as:

**social justice and equity, promoting and protecting better health for all, leaving no-one behind. [With] a resolute focus on tackling inequalities in health, including those driven by racism and discrimination.(**[**FPH**](https://www.fph.org.uk/what-is-public-health/#:~:text=is%20Public%20Health%3F-,Public%20health%20is%20the%20science%20and%20art%20of%20preventing%20disease,%2C%20leaving%20no%2Done%20behind.)**)**

We define advocacy as the championing of the above principles within your everyday work.

10. Based on your experience of working in each of the following settings, to what extent do you agree with the statement 'I feel/felt able to advocate for public health principles'? (*Likert scale per setting with options of: Strong agree, Agree, Disagree, Strong disagree, N/a*)

10.1. Local authority

10.2. NHS Trust

10.3. NHS England - regional

10.4. NHS England - national

10.5. OHID (or equivalent) - regional

10.6. OHID (or equivalent) - national

10.7. DHSC (or equivalent) - regional

10.8. DHSC (or equivalent) - national

10.9. UKHSA (or equivalent) - regional

10.10. UKHSA (or equivalent) - national

10.11. Other regional organisation (e.g. TfL)

10.12. International organisation (e.g. WHO)

10.13. VCSE

10.14. Academia

10.15. Other setting (please see Q11)

11. If you indicated another setting above, please expand below (*free text*)

12. Please give an overview of why you gave the answers above, including what factors enabled or inhibited you from advocating for public health principles in each setting. (*free text*)

13. In relation to your employment/placements, what barriers have you faced around advocacy? Please indicate all that apply. (*dropdown, multiple selections allowed*)

Employer/placement lack of understanding of Public Health Role

Lack of clarity around what is considered appropriate or acceptable within organisations

Perceived negative consequences of advocacy (e.g. job threat)

Employer concerns around politically sensitive issues

Other (please expand in Q16)

I have not experienced any barriers related to this

14. In relation to your own knowledge and confidence, what barriers have you faced around advocacy? Please indicate all that apply. (*dropdown, multiple selections allowed*)

Lack of personal confidence

Feeling issues fall outside of your remit (e.g. international issues)

Lack of knowledge of issues

Lack of mentorship/role models regarding advocacy

Lack of time

Other (please expand in Q16)

I have not experienced any barriers related to this

15. What practical issues have caused barriers to advocacy? Please indicate all that apply. (*dropdown, multiple selections allowed*)

Access to advocacy structures and organisations

Access to support (e.g. union support)

Access to advocacy training

Length of placements/employment (i.e. short timeframes leading to not feeling embedded)

Other (please expand in Q16)

I have not experienced any barriers related to this

16. If you have selected 'other' to any of the above please expand below (*free text*)

17. Are there any other avenues via which you advocate for public health principles which you would like to highlight? (*free text*)

18. Are there any courses / resources which have helped you advocate for public health principles that you would like to highlight? (These may be shared anonymously with public health practitioners) (*free text*)

19. Please indicate your gender below (*dropdown, single selection allowed)*

Female

Male

Non-binary

Prefer not to say

20. Please indicate your age bracket (*dropdown, single selection allowed)*

Under 25

26-30

31-35

36-40

41-45

46-50

51-55

56-60

61-65

66+

Prefer not to say

21. Please indicate your ethnicity below (*dropdown, single selection allowed)*

Asian or Asian British

Black, Black British, Caribbean or African

White

Mixed or multiple ethnic groups (please see Q22)

Other (please see Q22)

Prefer not to say

22. If you would like to provide any further details regarding your ethnicity selected above please do so here (*free text*)
