# Supplementary material for: Experiences of professional public health advocacy in the UK health and social care system: a qualitative survey study [using the Theoretical Domains Framework]
Source: J Public Health (Oxf). 2026 May 13;48(2):610–9. doi: 10.1093/pubmed/fdag035 (PMC13223560; doi:10.1093/pubmed/fdag035)
Supplement: JPH_Marynissen_et_al_appendix_2_supplementary_quotes_fdag035 [file jph_marynissen_et_al_appendix_2_supplementary_quotes_fdag035.docx]

Appendix 2: supplementary quotes for discussed themes

| **Theme** | **Quote** |
| --- | --- |
| Social and professional role: *Are PH practitioners seen as independent professionals or as service providers?* | |
| Advocacy as part of PH role | ‘[I]n general in LA or NHS Trust I feel that part of role is seen as tackling health inequalities and equity’  (P8, Registrar (medical or dental background), ST3)  ‘I felt had the ability to but not always permitted to as was not in the scope of work or responsibility’  (P17, Registrar (non-medical background), ST3)  ‘I don't think I've ever felt I can't advocate for PH principles- it's my job!’  (P22, Registrar (medical or dental background), ST5)  ‘I feel that it's part of my professional duty to advocate for public health principles […] I've never come across a situation where anyone has tried to stop me doing it - I assume that they expect me to do it as part of my professional role.’  (P26, Consultant, (medical or dental background), >5yrs post-CCT)  ‘Advocacy within my role in international organisation work was core to the work and clearly encouraged.’  (P45, Registrar (medical or dental background), ST2) |
| No room for advocacy in certain organisations (e.g. UKHSA) due to nature of the work (e.g. reactive, protocol-driven) | ‘My work in UKHSA was reactive and I do feel that some of their policies do not consider equity/health inequalities especially strongly. As it is quite protocol driven I am not sure I felt able to advocate differently.’  (P8, Registrar (medical or dental background), ST3)  ‘UKHSA - my role was health protection so it wasn't as relevant as my work only included acute health protection issues (not wider).’  (P58, Consultant (non-medical background), >5yrs post-CCT)  ‘UKHSA regional - whilst the nature of the work was entirely public health focused (health protection), I selected disagree because the nature of my role on the acute desk was very process/SOP/risk assessment led. The role itself didn't lend itself so much to getting involved in terms of principles like collaboration/joint-working and addressing health inequalities, or looking at wider determinants.’  (P7, Registrar (non-medical background), ST4)  ‘In UKSHA I felt much more pressure to focus on the job in front of me rather than making change in any meaningful way’  (P11, Consultant (non-medical background), <5yrs post-CCT)  ‘UKHSA HPT is an operational role not really doing wider system working as an StR’  (P19, Registrar (non-medical background), ST3)  ‘Health protection teams are disconnected - they have become a tick box exercise with no head space for wider public health considerations’  (P44, Registrar (medical or dental background), ST5) |
| NHS: healthcare focussed, immediate and acute pressures   - Though not ubiquitous experience | ‘Local NHS health boards have a tendency to focus on healthcare public health. Central government is heavily constrained by delivery focus, policy, protocol, programme for government, and ministerial priorities.’  (P3, Registrar (medical or dental background), ST4)  ‘In NHS Trusts, a level of concincing [sic] is required to do PH work, as it is not their first priority nor their main business.’  (P13, Consultant (non-medical background), <5yrs post-CCT)  ‘ICB lack of priority on population health/public health approach’  (P19, Registrar (non-medical background), ST3)  ‘Similarly, NHS trusts have their own hierarchies and priorities which may not always make PH a priority. It seems if you can create links between public health principles having mutual benefits to their own metrics and priorities there are also opportunities for advocacy.’  (P24, Registrar (medical or dental background), ST2)  ‘NHS Trust - although there is much more respect for professionalism and skills are more highly valued, competing acute priorities take precedence’  (P33, Consultant (medical or dental background), >5yrs post-CCT)  ‘I think in some places the way we worked as all guided by public health principles (e.g. LA). In places like UKHSA and ICB, they welcome PH principles but these probably aren't the top guiding force for why things are done the way they are (e.g. clinical guidelines, cost implications, NHS/DHSC requirements, etc.) so therefore more constrained environments where can't be directed as much.’  (P46, Registrar (non-medical background), ST3)  ‘I have been lucky to work in public health teams and environments that strongly advocate for these principles - local authority, NHS and OHID’  (P52, Practitioner (portfolio scheme) (non-medical background))  ‘In hospital settings, there is a general feeling that prevention is someone else, and not the responsibility of staff in hospital’  (P59, Practitioner (portfolio scheme) (non-medical background))  ‘NHS - More limited scope.  Genuine interest and appetite but rhetoric (i.e. shift to prevention, tackling inequalities) can be difficult to turn into reality at scale due to focus on immediate pressure and shorter term planning/outcomes.’  (P56, Consultant (portfolio scheme) (non-medical background), <5yrs post-CCT) |
| Academia can be difficult to advocate within academic rigour, narrow focus | ‘If it was a sliding scale I would say that there was less opportunities in academia compared to other locations not that it was absent.’  (P53, Registrar (non-medical background), ST5)  ‘Research work in academia can definitely advocate for and enact public health principles, although the need for academic rigour/thoroughness (e.g. in public health studies) can add burden which might hamper/stifle some public health principles (e.g. community engagement and collaboration). Also ‘in academia the work of on-the-ground public health practitioners was not always understood’  (P7, Registrar (non-medical background), ST4)  ‘In academia, I think sometimes the focus on principles can hinder practical progress - there is a certain amount purism that is very admirable but does not always translate to on the ground action in public health practice.’  (P11, Consultant (non-medical background), <5yrs post-CCT)  ‘academic public health can often take a narrow viewpoint due to the specialised nature of research, and teams are often lacking in diversity which is likely to have an impact given how research questions are identified, prioritised, and addressed.’  (P16, Registrar (non-medical background), ST3) |
| Higher level political organisations: geared towards ministerial priorities | ‘Local NHS health boards have a tendency to focus on healthcare public health. Central government is heavily constrained by delivery focus, policy, protocol, programme for government, and ministerial priorities.’  (P3, Registrar (medical or dental background), ST4)  ‘My experience of DHSC is that public health principles are very much secondary to ministerial priorities, and many civil servants see their primary responsibility as sophistry in support of ministerial priorities and public health values are rapidly reduced to rhetorical devices.’  (P9, Consultant (non-medical background), <5yrs post-CCT)  ‘Usually inhibited by work being steered by those less interested in public health principles (eg. ministers) or less ''able'' the work required to embed and prioritise public health principles often quoted as being due to acute pressing time sensitive issues (eg. NHS trusts)’  (P55, Registrar (medical or dental background), ST5) |
| Well-supported in Local Authority   - But there could be barriers with other departments | ‘Local authority PH I have found very much focused on public health principles.’  (P7, Registrar (non-medical background), ST4)  ‘In the local authority […] everyone had a good understanding of public health principles, so advocacy with external stakeholders was encouraged and considered part of the job’  (P10, Registrar (medical or dental background), ST2)  ‘Local authority: work in a Marmot city, so advocacy for public health principles is discussed frequently.  (P14, Registrar (non-medical background), ST2)  ‘. In LA I found that everyone is bought-in from public health officers all the way up to Director of Public health and found that many colleagues in other departments were also bought in and understand the principles of public health.’  (P51, Registrar (medical or dental background), ST4)  ‘Local authority advocacy is facilitated/hindered by dynamics and relationships with other key departments.’  (P39, Registrar (non-medical background), ST3) |
| Type of work | ‘Advocacy is part of my everyday work in my local authority setting. Perhaps this is a reflection of the higher level of seniority in this role compared to others where I felt the work was more project focussed.’  (P41, Consultant (medical or dental background), <5yrs post-CCT)  ‘UKHSA national role, it was still discussed frequently, but nature of the role meant that not as much action was possible.’  (P14, Registrar (non-medical background), ST2)  ‘[I]d [sic] not feel I could implement ph principles in the CSU as this work was responding to clients tenders so working to a contract and could not implement principles in these.’  (P37, Registrar (medical or dental background), ST2) |
| Lack of authority | ‘Lack of authority as a trainee’  (P4, Consultant (non-medical background), <5yrs post-CCT)  ‘Also the whole influencing without authority’  (P22, Registrar (medical or dental background), ST5) |
| Perceived role | ‘I am unsure to what extent I can access other external advocacy settings as a public health registrar. For example, there are advocacy organisations that I would want to approach but feel that I may be frowned upon as a public health representative because we are (or at least often perceived to be) somewhat aligned to the state or to government decision making. On the other hand, however, I feel that within the work/professional settings and communities which I have access to (local authority public health team, faculty of public health, BMA) that some of the causes I want to advocate for would be seen as politically risky and "too far to the left", for example.’  (P23, Registrar (medical or dental background), ST1)  ‘It helps when you are in an organisation that understands what public health is, what function and role PH has and why it is so important. There is a lot of misunderstanding’  (P2, Registrar (medical or dental background), ST4)  ‘Organisations are vague on advice / restrictions when specificity is required for effectiveness.’  (P40, Registrar (medical or dental background), ST5) |
|  |  |
| Environmental context and resources: *organisational structure and allocated resource affect perceived and actual ability to advocate for PH principles* | |
| Financial constraints: PH taking on other council functions / impacted ability to maintain PH focus | ‘[L]oss of grant funding to other council budgets, specialist analytical staff lost into corporate functions or left due to lack of value of technical skills, ph having to take on management of other council functions’  (P33, Consultant (medical or dental background), >5yrs post-CCT)  ‘[W]ithout a broader range of mandated public health services, and clearer guidance on the minimum expected service levels, the "ring-fenced" public health grant can get shifted more towards wider determinant/other council work to the detriment of core public health functions.’  (P40, Registrar (medical or dental background), ST5)  ‘I think the facilitating factors were political alignment, well-resourced public health teams’  (P42, Registrar (non-medical background), ST4) |
| Financial constraints: perceived inability to advocate for programmes /services as not economically viable, perception of advocacy=action | ‘[W]hat I can do is very limited by resources; UKHSA acute response team, due to resource limitation, we cannot do much and we also don’t have control over local authority budgets (an example would be financial support for families who need prolong exclusion for STEC E coli)’  (P6, Registrar (medical or dental background), ST3)  ‘In local authorities and NHS trusts, you have to be somewhat politically savvy if you want to make change - if you advocate too strongly and idealistically, you will be dismissed as not understanding the practical constraints of the situation’  (P11, Consultant (non-medical background), <5yrs post-CCT)  ‘Advocacy for local public health measures is impacted by the resourcing of local systems. With most teams having made substantial cuts in recent years to services, it can be difficult to advocate or initiate system action knowing that resources are already being diverted away from essential public health action’  (P40, Registrar (medical or dental background), ST5)  ‘[L]ack of resources in the organisation to make an impact’  (P6, Registrar (medical or dental background), ST3) |
| Organisational structure: when drawn into corporate/managerial roles, loss of opportunity for advocacy | ‘Local authority - loss of professionalism in public health, DPHs selected for corporate fit rather than PH skills, lack of independence and autonomy’  (P33, Consultant (medical or dental background), >5yrs post-CCT) |
| Organisational structure: where PH sits in relation to Exec / other functions | ‘The ability to advocate within the organisation, and within the local and national systems is heavily influenced by the structures and environment the Director of Public Health works in. DsPH that are directly accountable to the Chief Executive Officer Are better able to influence I think than those that report to other Directors; these DsPH can directly shape the council strategy and advocate for Public Health independently of other directorates’  (P40, Registrar (medical or dental background), ST5) |
| Politics | ‘The political environment of my local authority placement has allowed me to advocate for public health principles that I am interested in and care deeply about […] I am not sure this would have been well received or even allowed at different, adjacent councils with other political leanings.’  (P23, Registrar (medical or dental background), ST1)  ‘The local authorities I have worked in have had political make ups which meant in general there haven't been many ideological differences. In my experience public health advocacy and interventions are generally supported but small p politics can get in the way’  (P40, Registrar (medical or dental background), ST5)  ‘I think the facilitating factors were political alignment’  (P42, Registrar (non-medical background), ST4)  ‘In LA there are political barriers, as well as the need to work with partners, who have different perspectives, motivations and drivers, which you have to work with in order to get PH work done.’  (P13, Consultant (non-medical background), <5yrs post-CCT)  ‘[W]hile local authority provides breadth to advocate for public health principles across a wide range of settings, at times the "democratic" processes, bureaucracy, and political nature of the organisation make advocacy for public health principles challenging’  (P24, Registrar (medical or dental background), ST2)  ‘Political/professional expediency of public health leaders not challenging power’  (P47, Registrar (medical or dental background), ST5)  ‘In the LA our posts are politically restricted so we cant be publicly party political critical’  (P58, Consultant (non-medical background), >5yrs post-CCT)  ‘The negative aspects are related to observed hesitation from colleagues to engage where the issue is politically sensitive.’  (P18, Registrar (non-medical background), ST2) |
| Organisational culture | ‘Having an environment that is open to ideas and discussions is an important factor in promoting or allowing for advocacy in public health.’  (P32, Consultant (medical or dental background), >5yrs post-CCT)  ‘Organisational atmosphere’  (P38, Registrar (non-medical background), ST5) |
| Team capacity | ‘There doesn't seem to be enough time or capacity to focus on more strategic and preventative work which is the major barrier to advocacy’  (P42, Registrar (non-medical background), ST4) |
|  |  |
| Social influences: *alignment of values is key for effective advocacy* | |
| Importance of supportive teams   - Aligned values - May struggle to feel part of team on short-term placements - Role models | ‘[G]enerally not in organisations long enough to influence or advocate for long term change as relationship building and change takes time which on the training scheme we are short of.’  (P55, Registrar (medical or dental background), ST5)  ‘Worked in supportive teams with values aligned with the above definition (sometimes these were implicit and not always written down in the same way).’  (P64, Registrar (non-medical background), ST3)  ‘It can be challenging as an outsider (supernumerary PH trainee joining teams on a temporary basis) to advocate and influence colleagues’  (P16, Registrar (non-medical background), ST3)  ‘[S]hort timeframes leading to not feeling embedded is a big issue’  (P17, Registrar (non-medical background), ST3)  ‘The positive aspects is that the colleagues in the Public Health and communities team are tuned in and enthusiastic about everything to do with public health which makes it easier to advocate for public health principles’  (P18, Registrar (non-medical background), ST2)  ‘I also have well established activists in my public health team who I can look for their vocal, longstanding and admirable work.’  (P23, Registrar (medical or dental background), ST1)  ‘As a team we share collective values and support each other in trying to implement these’  (P27, Consultant, (medical or dental background), >5yrs post-CCT)  ‘Lack of support from colleagues who don’t fight the fight with you.’  (P1, Registrar (non-medical background), ST4)  ‘Even when there is buy-in from senior leaders where more junior colleagues are not bought-in they become a practical blocker to change.’  (P51, Registrar (medical or dental background), ST4) |
| Importance of supportive leadership   - Also impacts confidence to raise issues in the first place (**beliefs about capabilities**) - Psychological safety (**emotion**) | ‘My ability to advocate for public health principles has been influenced a lot by the leadership/organisational culture of the placements.’  (P40, Registrar (medical or dental background), ST5)  ‘There was a strong strategic direction towards reducing health inequalities that didn't seem to be noticeably undermined. This could be due to strong, credible leadership at the top of the Directorate.’  (P42, Registrar (non-medical background), ST4)  ‘I think the facilitator here might have been my project supervisor who has been an articulate and enthusiastic advocate for the public health skillset (even though she is not public health trained) which has created equal enthusiasm and a respect for the the [sic] expertise from the rest of the team’  (P42, Registrar (non-medical background), ST4)  ‘[G]etting [public health principles] meaningfully communicated for change to those in more authoritative leadership roles and them to act on these is a challenge’  (P43, Registrar (medical or dental background), ST2)  ‘Many senior public health figures seem not to understand the importance of agenda setting and wait for political direction on their agendas and remits.’  (P47, Registrar (medical or dental background), ST5)  ‘Inhibitors: Defensive or brusque leadership styles’  (P49, Registrar (non-medical background), ST5)  ‘I am no longer managed by a public health consultant, and my DD is a career civil servant who has been very negative about the profession. I have tried to advocate for public health and been told I am 'undermining team cohesion' and 'impacting colleagues' confidence' when taking a public health approach.’  (P50, Practitioner (portfolio scheme) (non-medical background))  ‘I feel like there is a need for employer and professional support when advocating for issues. I have felt that this is often lacking, particularly when the advocacy is related to employment terms and conditions or the work that the organisation focuses on. ‘  (P61, Registrar (non-medical background), ST4) |
| Training programme bias and effects | ‘I think the training programme has in recent years been guilty of differential treatment of registrars / favouring certain types of practitioners […] these issues also create problems in the types of leaders created and promoted; advocacy requires bravery and tenacity, which comes from confidence and supportive settings leading to capacity and resilience.’  (P40, Registrar (medical or dental background), ST5) |
|  |  |
| Skills: *need for training* | |
| Advocacy training needed, including embedding within training programme   - Not felt by all | ‘[I]t would be nice to receive formal advocacy training.’  (P10, Registrar (medical or dental background), ST2)  ‘Enablers - professional training’  (P29, Registrar (non-medical background), ST3)  ‘Training programmes do not prepare registrars for working within these systems, instead putting the onus on StRs to "reflect on system leadership" with little guidance or tuition.  (P40, Registrar (medical or dental background), ST5)  ‘I feel that my experience and training has enabled me to advocate in all settings, whether people agree with me or not and whether my opinion is asked for’  (P54, Consultant (portfolio scheme) (non-medical background), <5yrs post-CCT)  ‘I have the freedom to advocate and influence and as I've worked (prior to PH) in other public bodies and the VCS sector, I know how to raise the principles as it relates to their work.’  (P62, Consultant (non-medical background), >5yrs post-CCT) |
| Influencing as skill   - ‘selling importance’ | ‘Enabling: role model, clarity of vision that included PH principles, being able to 'sell' importance’  (P36, Registrar (medical or dental background), ST3)  ‘There is no interest in public health in my division of DHSC. Even when CMO or Ministers request information on our topic, the briefings provided are poor, often based on authors' personal opinions.’  (P50, Practitioner (portfolio scheme) (non-medical background)) |
|  |  |
| Goals: *importance of clarity* | |
| Clarity of objective/vision as an enabler to advocacy | ‘Enabling: role model, clarity of vision that included PH principles, being able to 'sell' importance’  (P36, Registrar (medical or dental background), ST3)  ‘Inhibitors: Defensive or brusque leadership styles, lack of clarity regarding objectives or involvement of the team in setting these, organisational change and uncertainty, lack of morale and capacity in key partners’  (P49, Registrar (non-medical background), ST5) |
|  |  |
| Knowledge: *knowledge of PH principles within wider team and org influence ability to advocate* | |
| Understanding of PH principles amongst wider team is helpful, converse also true   - Covid shone a light on PH principles - Shared understanding of principles | ‘Generally, I think the pandemic gave a renewed focus on inequalities, racism, and the health of communities to a wider range of local authority partners and teams. Health genuinely became everyone's business’  (P63, Registrar (non-medical background), ST1)  ‘[M]ore challenges working with health services as the focus has been less around targeted approaches and there are still challenges in shifting the focus upstream. The way health describes prevention is different to how public health often describes it (primary vs secondary prevention)  (P52, Practitioner (portfolio scheme) (non-medical background))  ‘[E]veryone had a good understanding of public health principles, so advocacy with external stakeholders was encouraged and considered part of the job.’  (P10, Registrar (medical or dental background), ST2)  ‘I note senior leaders understand the principles and are bought-in whereas more junior colleague civil servants do not have an in-depth understanding of health and public health principles in order to complete tasks in a meaningful way.’  (P51, Registrar (medical or dental background), ST4) |
|  |  |
| Beliefs about consequences | |
| Negative consequences / impact on training | ‘[T]here is a degree of personal challenges to advocacy and the position this can leave you in within a board’  (P45, Registrar (medical or dental background), ST2)  ‘Racism is also prevalent in hospital, and is not given the importance it deserves, especially by middle managers who don't want to rock the boat’  (P59, Practitioner (portfolio scheme) (non-medical background)) |
| Need to be politically savvy | ‘In local authorities and NHS trusts, you have to be somewhat politically savvy if you want to make change - if you advocate too strongly and idealistically, you will be dismissed as not understanding the practical constraints of the situation’  (P11, Consultant (non-medical background), <5yrs post-CCT) |
|  |  |
| Emotion |  |
| Psychological safety | ‘[I]n these more open and collaborative settings, factors such as real spaces for dialogue, or more psychological safety lead to better advocacy for Public Health.’  (P40, Registrar (medical or dental background), ST5)  ‘Enablers: Supportive teams and line managers, environments that encourage asking questions’  (P49, Registrar (non-medical background), ST5) |
| Fear of being viewed as a dissident, harming career progression | ‘I've worked in regional teams where I think there is real fear of being viewed as a dissident and the subsequent loss of power/influence/jobs. In private regional leaders will say they are advocating as best they can but this atmosphere can stifle passion, creative/critical thinking, and engagement with work at all levels of the organisation’  (P40, Registrar (medical or dental background), ST5) |
| Lack of morale / capacity amongst partners | ‘Inhibitors: Defensive or brusque leadership styles, lack of clarity regarding objectives or involvement of the team in setting these, organisational change and uncertainty, lack of morale and capacity in key partners’  (P49, Registrar (non-medical background), ST5) |
| Confidence / tolerance of discomfort | ‘Advocacy (and to a greater extent activism) requires a certain degree of personal discomfort, professional vulnerability/risk, cultural humility, and a continuing process of unlearning - these are things that senior public health figures and establishments have no appetite for.’  (P47, Registrar (medical or dental background), ST5) |
